# The HBP Pathway Inhibitor FR054 Enhances Temozolomide Sensitivity in Glioblastoma Cells by Promoting Ferroptosis and Inhibiting O‐GlcNAcylation

**DOI:** 10.1111/cns.70546

**Published:** 2025-08-07

**Authors:** Rongxu Ye, Wanghao Zhang, Huayang Zhang, Shanqiang Qu, Junyi Xu, Rongyang Xu, Ye Zhu, Guanglong Huang, Xi‐an Zhang, Guo‐zhong Yi

**Affiliations:** ^1^ Department of Neurosurgery Nanfang Hospital, Southern Medical University Guangzhou Guangdong People's Republic of China; ^2^ The Laboratory for Precision Neurosurgery, Nanfang Hospital Southern Medical University Guangzhou Guangdong People's Republic of China; ^3^ The First Clinical Medical College, Southern Medical University Guangzhou Guangdong People's Republic of China; ^4^ School of Public Health Southern Medical University Guangzhou Guangdong People's Republic of China; ^5^ Nanfang Glioma Center Guangzhou Guangdong People's Republic of China; ^6^ Institute of Brain Disease, Nanfang Hospital, Southern Medical University Guangzhou Guangdong People's Republic of China

**Keywords:** ferroptosis, glioblastoma, hexosamine biosynthesis pathway inhibitor FR054, O‐GlcNAcylation, temozolomide sensitivity

## Abstract

**Background:**

The clinical efficacy of temozolomide (TMZ) in glioblastoma (GBM) patients is often limited by the development of resistance. To date, no clinically validated therapeutic strategies exist to restore sensitivity to TMZ treatment. In this study, we investigated the potential of FR054, a hexosamine biosynthesis pathway (HBP) inhibitor, to sensitize GBM cells to TMZ and elucidated its underlying molecular mechanism.

**Methods:**

TMZ‐resistant U87‐MG and A172 cell lines were generated through stepwise exposure to increasing concentrations of TMZ. Proteomics and bioinformatics analyses revealed HBP activation in these resistant cells. The effects of FR054 alone or in combination with TMZ were assessed using cell line models, GBM organoid models, and intracranial xenograft models. Transcriptomic analysis and validation experiments were further conducted to explore the molecular mechanisms involved.

**Results:**

Long‐term exposure to TMZ induced resistance in U87‐MG and A172 GBM cells, which was associated with the activation of the HBP pathway. PGM3, a key enzyme in the HBP, was found to correlate with poor prognosis in GBM patients. The combination of FR054, a specific PGM3 inhibitor, with TMZ exhibited synergistic inhibitory effects in vitro and superior inhibitory efficacy in GBM organoid models. In vivo, this combination significantly suppressed tumor progression and prolonged survival in orthotopic xenograft mice with minimal side effects. Mechanistically, FR054 enhanced TMZ sensitivity by inhibiting protein O‐GlcNAcylation and promoting ferroptosis via the upregulation of HMOX1 and downregulation of GPX4.

**Conclusion:**

Our findings demonstrate that targeting the HBP pathway with FR054 can overcome TMZ resistance in GBM by reducing O‐GlcNAc modification and inducing ferroptosis. This novel approach enhances the efficacy of TMZ, offering a promising therapeutic strategy for GBM patients with limited treatment options.

## Graphical abstract

Targeting the HBP pathway with FR054 can effectively overcome TMZ resistance in GBM by reducing O‐GlcNAc modification levels and inducing ferroptosis, thereby presenting a promising therapeutic strategy for GBM patients who have limited treatment options.

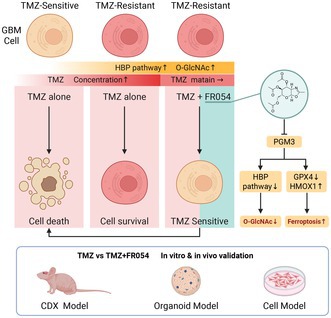

## 1 Introduction

Glioblastoma (GBM) is the most aggressive primary malignant brain tumor in adults, characterized by genetic heterogeneity, rapid growth, and non‐specific symptoms [1]. Despite therapeutic advances, including maximal safe surgical resection followed by radiotherapy and temozolomide (TMZ) chemotherapy, the prognosis remains poor, with a median survival of about 15 months and a five‐year survival rate under 10% [2]. The highly infiltrative nature of GBM limits complete resection, necessitating adjuvant therapies to manage recurrence and improve outcomes.

TMZ, an oral alkylating agent, is pivotal in GBM treatment [3]. It works through hydrolysis to form methyltriazenoimidazole carboxamide (MTIC), which methylates DNA, leading to DNA damage and apoptosis. Despite its effectiveness, most GBM patients develop resistance to TMZ, resulting in tumor recurrence [4]. The primary mechanism of TMZ resistance involves the DNA repair enzyme O^6^‐methylguanine‐DNA methyltransferase (MGMT), with MGMT promoter methylation generally correlating with a better response to TMZ [5]. However, many patients remain resistant, indicating a complex interplay of factors. Currently, there are no clinically approved drugs to reverse TMZ resistance, underscoring the need for new therapeutic strategies.

The hexosamine biosynthesis pathway (HBP) is a critical glucose‐derived metabolic route, accounting for approximately 2%–3% of total glucose uptake in cells. This pathway begins with fructose‐6‐phosphate and glutamine, progressing through a series of enzymatic reactions to produce uridine diphosphate‐N‐acetylglucosamine (UDP‐GlcNAc). UDP‐GlcNAc is essential for maintaining the structural integrity of cells and tissues by serving as a substrate for the synthesis of chitin, peptidoglycan, glycosaminoglycans, and glycosylphosphatidylinositol (GPI) anchors, which anchor cell surface proteins to the plasma membrane. Key enzymes such as GFAT1/GFAT2, GNPNAT1, PGM3, and UAP1 are frequently upregulated in various cancers, correlating with increased UDP‐GlcNAc production and global O‐GlcNAcylation [6]. These changes support tumor growth, metastasis, and therapeutic resistance through mechanisms including enhanced glycosylation of growth factor receptors (e.g., EGFR), metabolic reprogramming, and activation of oncogenic signaling pathways. Notably, oncogenic KRAS, hypoxia, and metabolic stress further drive HBP activation. Inhibition of HBP enzymes, particularly GFAT1/2, suppresses cancer cell proliferation and enhances immune responses by reducing extracellular matrix components like hyaluronan. Targeting HBP also sensitizes tumors to immunotherapy and phagocytosis [7]. These findings highlight the HBP as a promising therapeutic target in cancer, with potential for combination therapies targeting both tumor metabolism and immunity. Dysregulation of these processes has been implicated in various cancers, although their specific roles in GBM remain underexplored.

Moreover, UDP‐GlcNAc serves as a substrate for several glycosylation processes, including O‐linked β‐N‐acetylglucosamine glycosylation (O‐GlcNAcylation). O‐GlcNAcylation, dynamically regulated by O‐GlcNAc transferase (OGT) and O‐GlcNAcase (OGA), involves the addition or removal of N‐acetylglucosamine (GlcNAc) moieties on serine or threonine residues of proteins, thereby modulating their post‐translational states. This modification is influenced by nutrient availability, OGT/OGA enzyme activity, and intracellular UDP‐GlcNAc levels. Notably, elevated O‐GlcNAc levels have been reported in various tumors, affecting diverse cellular functions such as proliferation, immune activation, apoptosis, stress responses, protein trafficking, and nutrient sensing [7, 8].

PGM3, a metabolic enzyme within HBP, has recently been implicated in various cancers [9, 10]. FR054, a competitive inhibitor of PGM3, was initially developed for breast cancer therapy [11]. Previous studies have demonstrated that FR054 induces growth arrest and apoptosis in cancer cells, reduces N‐ and O‐glycosylation, activates the unfolded protein response, and increases the accumulation of intracellular reactive oxygen species (ROS) in models of breast cancer, pancreatic cancer, and pancreatic ductal adenocarcinoma [11, 12, 13]. In pancreatic cancer, FR054 has also been shown to enhance sensitivity to gemcitabine, improving therapeutic efficacy [12]. Despite these promising findings in other cancers, there are currently no reports investigating the effects of FR054 in GBM.

In this study, we identified FR054 as a potential sensitizer for TMZ through bioinformatics approaches, a finding subsequently validated in multiple GBM cell lines. Our results were further corroborated in organoid models and intracranial orthotopic human GBM models. Transcriptomic sequencing revealed that the synergistic effects of FR054 and TMZ are associated with ferroptosis, a point confirmed by subsequent experiments. This research underscores the potential of targeting HBP and O‐GlcNAcylation as novel strategies to overcome TMZ resistance and improve outcomes in GBM patients. These findings provide new insights into developing more effective therapeutic approaches for GBM.

## 2 Materials and Methods

### 2.1 Cell Line and GBM Samples

The U87‐MG and A172 cell lines used in this study were purchased from the American Type Culture Collection (ATCC). GBM001 cells were primary GBM cells isolated from patient‐derived GBM tissue. All GBM samples were obtained from the Department of Neurosurgery, Nanfang Hospital. The GBM cells were cultured in Dulbecco's Modified Eagle Medium (DMEM, Gibco, 12491015) supplemented with 10% fetal bovine serum (FBS, Wuhan Pricella Biotechnology, 164210‐50) and 1% penicillin–streptomycin (New Cell & Molecular Biotech, C100C5). Cells were maintained at 37°C in a humidified incubator with 5% CO_2_.

### 2.2 In Vivo Xenograft Assay

For the in vivo xenograft assay, animal experiments were conducted following protocols approved by the Institutional Animal Care and Use Committee (IACUC) of Southern Medical University, in compliance with national guidelines for the care and use of laboratory animals. Briefly, 4–6‐week‐old female nude mice (BALB/c background, purchased from Sibeifu Biotechnology Co. Ltd.) were used for tumor implantation. U87‐MG‐luc cells (approximately 5 × 10^6^ cells suspended in 100 μL of PBS) were stereotactically injected into the right flank of each nude mouse at a position 2 mm lateral, 1 mm anterior, and 3.5 mm deep relative to the fontanel, ensuring consistent cell delivery and reproducibility of tumor formation. Intracranial injections and skin suture closure were carried out as previously described [14].

Tumor formation was monitored 10 days post‐implantation using an in vivo imaging system (Bruker, FX Pro) under isoflurane anesthesia to confirm successful engraftment. Mice were randomly assigned to different treatment groups after confirming tumor establishment. Throughout the study, tumor growth was monitored regularly using bioluminescence imaging. All procedures involving animals were approved by the Animal Research Ethics Committee of Southern Medical University and adhered strictly to the National Guidelines for the Care and Use of Laboratory Animals. For intracranial tumor growth monitoring, imaging was performed using the same small‐animal imaging facility under isoflurane anesthesia.

### 2.3 Organoid Construction and Culture

GBM organoids were established from tumor tissues collected during surgery. The excised tissues were immediately placed in cold preservation medium and transported back to the laboratory within 30 min on ice to ensure tissue viability. GBM tissues were then processed for organoid culture by mincing them into small fragments and seeding these fragments onto six‐well ultra‐low attachment plates. The plates were incubated in a humidified incubator at 37°C, with 5% CO_2_ and 21% O_2_, and maintained under continuous orbital shaking at 100 rpm to promote organoid formation and growth. The protocol for constructing GBM organoids was referenced as previously described in the literature [15].

The culture medium for short‐term maintenance of GBM organoids was changed every 48 h to ensure optimal nutrient supply and waste removal, supporting the sustained growth and health of the organoids. This method allows for the effective establishment and propagation of GBM organoids, providing a robust in vitro model for further experimental investigations.

### 2.4 Detection of Ferroptosis

To evaluate ferroptosis in GBM cells following treatment, several key indicators were assessed. Glutathione (GSH) levels were quantified using the GSH Assay Kit (Dojindo, G263). Intracellular labile iron was detected by employing FerroOrange (Dojindo, F374), with fluorescence intensity serving as the quantification parameter. Reactive oxygen species (ROS) levels were evaluated using the ROS Assay Kit (Dojindo, R252). All assays were conducted in triplicate and strictly adhered to the manufacturers' protocols to ensure accurate evaluation of ferroptosis‐related alterations.

### 2.5 Bioinformatics and Database Analysis

Gene Ontology Biological Process (GO‐BP) and Kyoto Encyclopedia of Genes and Genomes (KEGG) pathway enrichment analyses were performed using Database for Annotation, Visualization and Integrated Discovery (DAVID). Data from the Chinese Glioma Genome Atlas (CGGA) [16] (mRNAseq_325 [17, 18, 19] and mRNAseq_693 [20, 21]) were merged for comprehensive analysis. The University of California Santa Cruz (UCSC) [22] Xena platform was used to analyze TCGA_GBM and TCGA_LGG datasets. Survival analysis was conducted with the survminer [23] R package. Differential gene expression between tumor and normal tissues was evaluated using Gene Expression Profiling Interactive Analysis (GEPIA) [24]. Correlations between gene expression and the drug half‐maximal inhibitory concentration (IC50) values were analyzed using the Cancer Therapeutics Response Portal version 2 (CTRP2) [25, 26, 27] and Genomics of Drug Sensitivity in Cancer (GDSC) [28] databases. Differences in gene expression between tumor cells and other cell types were assessed using the Tumor Immune System Interactions in Single Cell Hub (TISCH) [29, 30] and the Gene Expression Omnibus (GEO) [31] dataset GSE84464 [32]. Drug‐target binding site predictions were performed using CB‐Dock2 [33]. SynergyFinder [34] quantified drug combination synergies. Gene set enrichment analysis (GSEA) explored correlations between gene expression and enriched pathways. Ferroptosis‐related gene information was retrieved from FerrDb [35].

### 2.6 Statistical Analysis

Experimental data are presented as mean ± standard deviation (SD) and were analyzed using GraphPad Prism 8.0.2 software. All datasets were tested for normality using the Shapiro–Wilk test. For comparisons between two groups, Student's *t*‐test was applied if the data followed a normal distribution; otherwise, the non‐parametric Mann–Whitney *U* test was used. For analyses involving more than two groups, one‐way or two‐way analysis of variance (ANOVA) was performed, followed by Tukey's multiple comparisons test for post hoc analysis. Survival analyses were conducted using the Kaplan–Meier (K–M) method, and differences in survival curves were evaluated by the log‐rank test. Correlations between gene expression levels and drug sensitivity were assessed using Spearman's rank correlation coefficient. Statistical significance was defined as *p* < 0.05 (More supporting information can be found in Data S1).

## 3 Results

### 3.1 Proteomics Analysis Reveals Enrichment of the Hexosamine Biosynthesis Pathway and Upregulation of O‐GlcNAcylation in TMZ‐Resistant Cells

**FIGURE 1:**
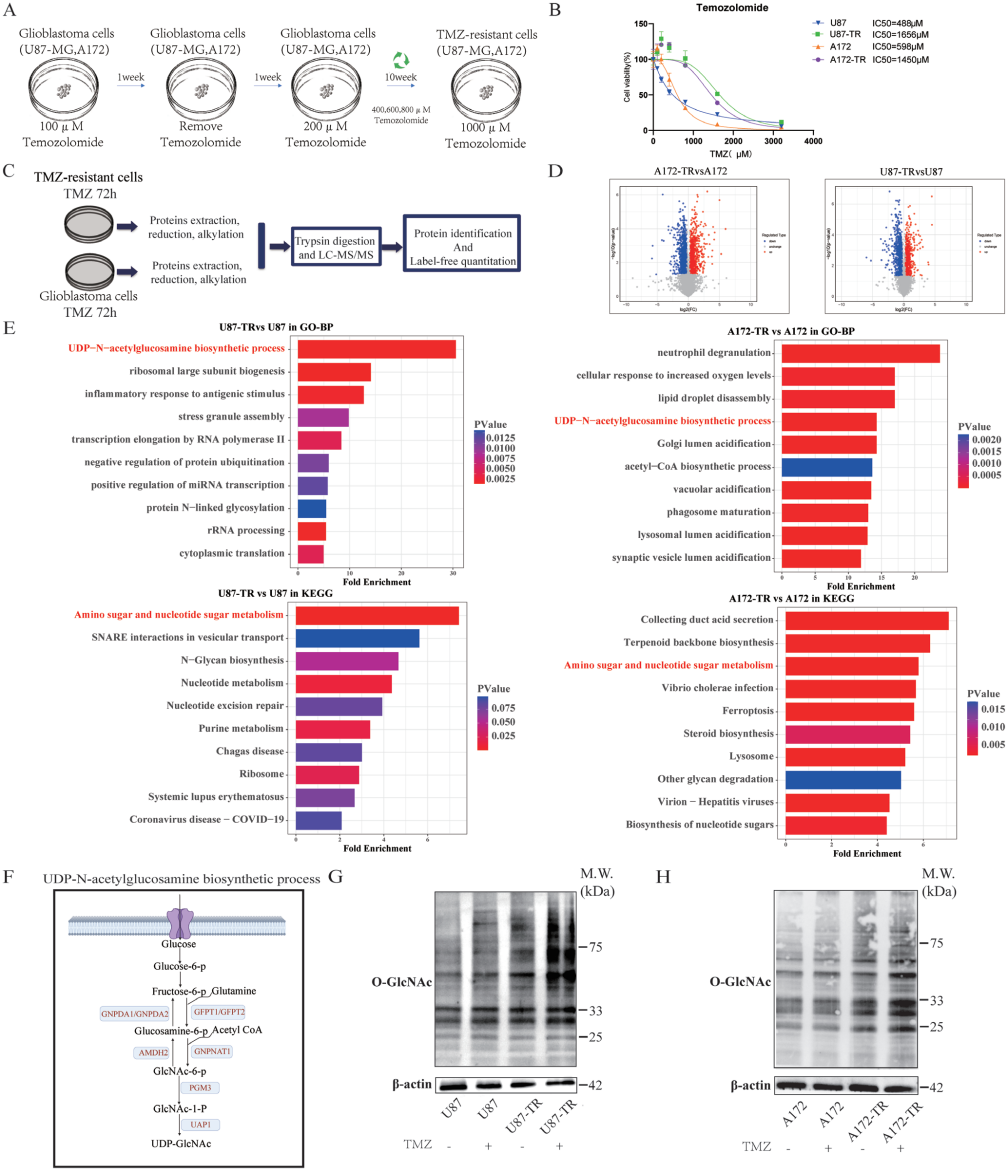
Proteomics analysis reveals enrichment of the hexosamine biosynthesis pathway and upregulation of O‐GlcNAcylation in temozolomide‐resistant cells. (A) U87‐MG and A172 cells were subjected to gradually increasing TMZ concentrations over approximately 10 weeks to induce resistance. (B) The IC50 values of TMZ‐resistant strains were approximately two‐fold higher than those of parental cells, confirming significant acquired resistance. (C) Schematic workflow of protein extraction and mass spectrometry analysis performed on TMZ‐resistant strains and GBM cell lines. (D) Volcano plot of differential protein expression. Proteins with fold change (FC) > 1.5 and *p* < 0.05 were defined as upregulated; FC < 0.67 and *p* < 0.05 as downregulated. (E) Bar plots showing the top 10 enriched GO‐BP and KEGG pathways for upregulated genes in U87‐TR vs. U87 (left) and A172‐TR vs. A172 (right), ranked by enrichment scores. Color intensity reflects statistical significance (*p*‐value). (F) Schematic representation of the hexosamine biosynthetic pathway (HBP), illustrating the enzymatic conversion of glucose and glutamine into UDP‐GlcNAc. (G) Western blot analysis of O‐GlcNAc expression in U87 and U87‐TR cells with or without 72‐h stimulation by 400 μM TMZ. (H) Western blot analysis of O‐GlcNAc expression in A172 and A172‐TR cells with or without 72‐h stimulation by 400 μM TMZ.

To investigate the mechanisms underlying TMZ resistance, GBM cell lines U87‐MG and A172 were utilized to establish TMZ‐resistant strains. The initial treatment concentration of TMZ was set at 100 μM for 1 week, followed by cycles of drug withdrawal, recovery, and reintroduction at incrementally higher concentrations (up to 1000 μM) over 10 weeks, mimicking clinical resistance development [36] (Figure 1A). After establishing resistant strains, a CCK‐8 assay demonstrated that prolonged TMZ treatment significantly increased the IC50 values of U87‐MG from 488 to 1656 μM and A172 from 598 to 1450 μM (Figure 1B), indicating reduced drug sensitivity and potential resistance development.

Furthermore, proteomics analysis was conducted on GBM cells, comparing TMZ‐resistant cell lines (A172‐TR and U87‐TR) with their non‐resistant parental counterparts (A172 and U87), with three replicates per group. Both groups were treated with 100 μM TMZ for 72 h, after which total proteins were extracted and analyzed via mass spectrometry (Figure 1C). Differential protein expression analysis identified 476 upregulated and 635 downregulated proteins in A172‐TR compared to A172, and 301 upregulated and 378 downregulated proteins in U87‐TR compared to U87. These differences were visualized using volcano plots (Figure 1D). Gene enrichment analysis focused on GO‐BP and KEGG pathways revealed that for U87‐TR/U87, top enriched pathways included UDP‐N‐acetylglucosamine biosynthetic process and amino sugar and nucleotide sugar metabolism (Figure 1E). Similarly, for A172‐TR/A172, significant enrichments were observed in these two pathways, suggesting a critical role for these metabolic processes in TMZ resistance.

Previous studies have demonstrated that the hexosamine biosynthesis pathway (HBP) culminates in the production of uridine diphosphate N‐acetylglucosamine (UDP‐GlcNAc) (Figure 1F), which serves as a substrate for O‐GlcNAcylation mediated by OGT. Conversely, OGA removes GlcNAc groups from proteins (Figure S1). Western blot analyses showed that 400 μM TMZ treatment for 72 h led to significant upregulation of protein O‐GlcNAcylation levels in both U87 and U87‐TR cells (Figure 1G). Notably, U87‐TR cells exhibited higher O‐GlcNAcylation levels than untreated controls, both before and after TMZ exposure. Similar trends were observed in A172 and A172‐TR cells (Figure 1H), suggesting that O‐GlcNAcylation plays a crucial role in TMZ‐treated GBM cells and their resistant variants.

### 3.2 High Expression of PGM3 Correlates With Poor Prognosis and TMZ Resistance in GBM Patients

**FIGURE 2:**
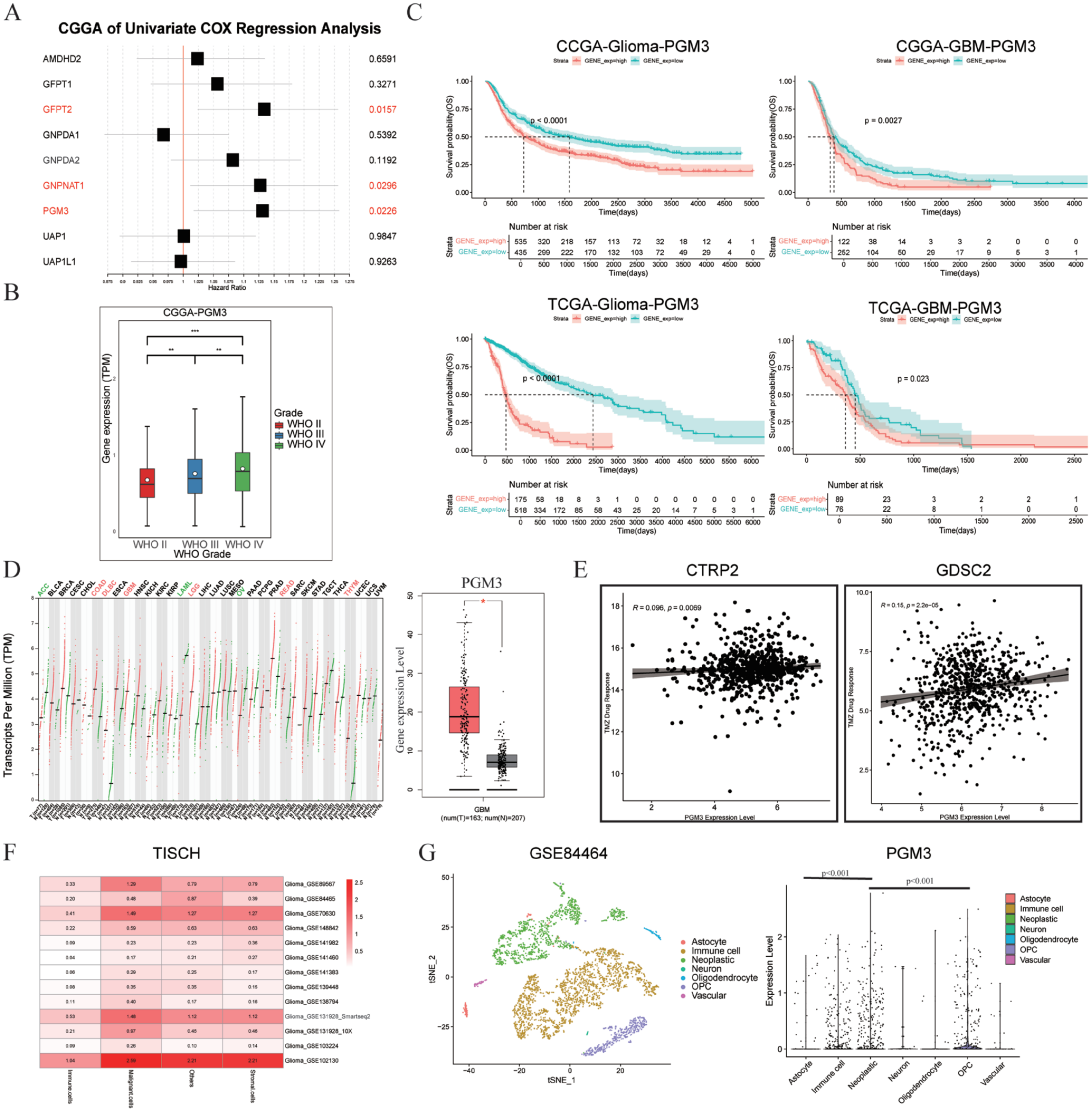
High expression of PGM3 correlates with poor prognosis and temozolomide resistance in GBM patients. (A) Univariate Cox regression analysis of CGGA GBM patient data demonstrates that high expression levels of GFPT2, GNPNAT1, and PGM3 are significantly associated with poor prognosis. (B) Bar plot illustrating PGM3 expression across WHO grades in CGGA glioma patients. (C) Kaplan–Meier survival analyses based on PGM3 expression levels in CGGA and TCGA datasets reveal significantly worse prognosis for patients with high PGM3 expression (Log‐rank *p* < 0.0001 for CGGA Glioma; *p* = 0.0027 for CGGA GBM; *p* < 0.0001 for TCGA Glioma; *p* = 0.023 for TCGA GBM). (D) Left: Comparison of PGM3 expression between tumor and normal tissues across 33 cancer types; Right: Significant upregulation of PGM3 in GBM compared to normal brain tissue (*p < 0.01). (E) Left: Scatter plot showing the correlation between PGM3 expression and temozolomide sensitivity in CTRPv2 (Spearman's *R* = 0.096, *p* = 0.0069); Right: Similar analysis in GDSC (Spearman's *R* = 0.15, *p* < 0.001). (F) Heatmap depicting PGM3 expression across different cell types in 13 glioma single‐cell sequencing datasets from TISCH. (G) Left: Scatter plot of cell clustering following dimensionality reduction and clustering annotation in the GSE84464 dataset; Right: Boxplot showing PGM3 expression levels in various cell types within GSE84464, with statistical significance determined by the KW test.

Further, we investigated the potential association between the HBP pathway and temozolomide resistance in GBM, as well as evaluated the relationship between key HBP enzymes and clinical outcomes. We integrated two major mRNA sequencing datasets (mRNAseq_325 and mRNAseq_693) from the CGGA, establishing a cohort of 374 GBM patients after excluding those with incomplete survival information. Using univariate Cox proportional hazards regression models, we found that high expression levels of GFPT2, GNPNAT1, and PGM3 were significantly associated with poorer overall survival (OS) in these patients (*p* < 0.05, Figure 2A). These enzymes may serve as critical factors contributing to unfavorable treatment responses in GBM.

Next, we performed WHO grading‐based correlation analysis using all glioma patient samples (*n* = 970) from CGGA (Figure 2B). PGM3 expression was significantly higher in WHO grade III compared to grade II patients (*p* < 0.01) and in WHO grade IV compared to grade III patients (*p* < 0.01), indicating a potential association between PGM3 expression and glioma grading. Additionally, we integrated RNA‐seq datasets from TCGA‐Glioma (from UCSC‐Xena, *n* = 693) and CGGA‐Glioma (*n* = 970) for Kaplan–Meier survival analysis using the survminer R package. Stratifying patients by PGM3 expression levels revealed that high PGM3 expression was significantly associated with worse clinical outcomes in both overall glioma patients and a subset of GBM patients (Figure 2C). Using the GEPIA database, which integrates TCGA tumor and GTEx normal tissue transcriptomic data, we analyzed 33 cancer types. PGM3 expression was significantly higher in tumor tissues compared to normal tissues in COAD, DLBC, GBM, LGG, READ, and THYM, while lower in ACC, AML, and OV, suggesting an oncogenic role for PGM3 in GBM (Figure 2D).

To explore the relationship between PGM3 expression and temozolomide sensitivity, we utilized the CTRPv2 and conducted a Spearman correlation analysis. The results indicated a positive correlation between PGM3 expression and temozolomide IC50 values (*R* = 0.096, *p* = 0.0069), suggesting reduced temozolomide sensitivity with high PGM3 expression. This finding was validated using the Genomics of GDSC dataset, which also showed a significant positive correlation (*R* = 0.15, *p* < 0.001) (Figure 2E). Finally, we used the TISCH to analyze single‐cell transcriptomic data from 13 glioma datasets. PGM3 expression was significantly higher in tumor cells compared to immune, stromal, and other cell types (Figure 2F). Further validation using the GSE84464 dataset from GEO confirmed these findings through Seurat‐based dimensionality reduction and clustering analysis (Figure 2G). Our results suggest that PGM3 is a potential therapeutic target in glioma.

### 3.3 Combination Therapy of TMZ and HBP Pathway Inhibitor FR054 Displayed a Significant Synergistic Effect on GBM Cells

**FIGURE 3:**
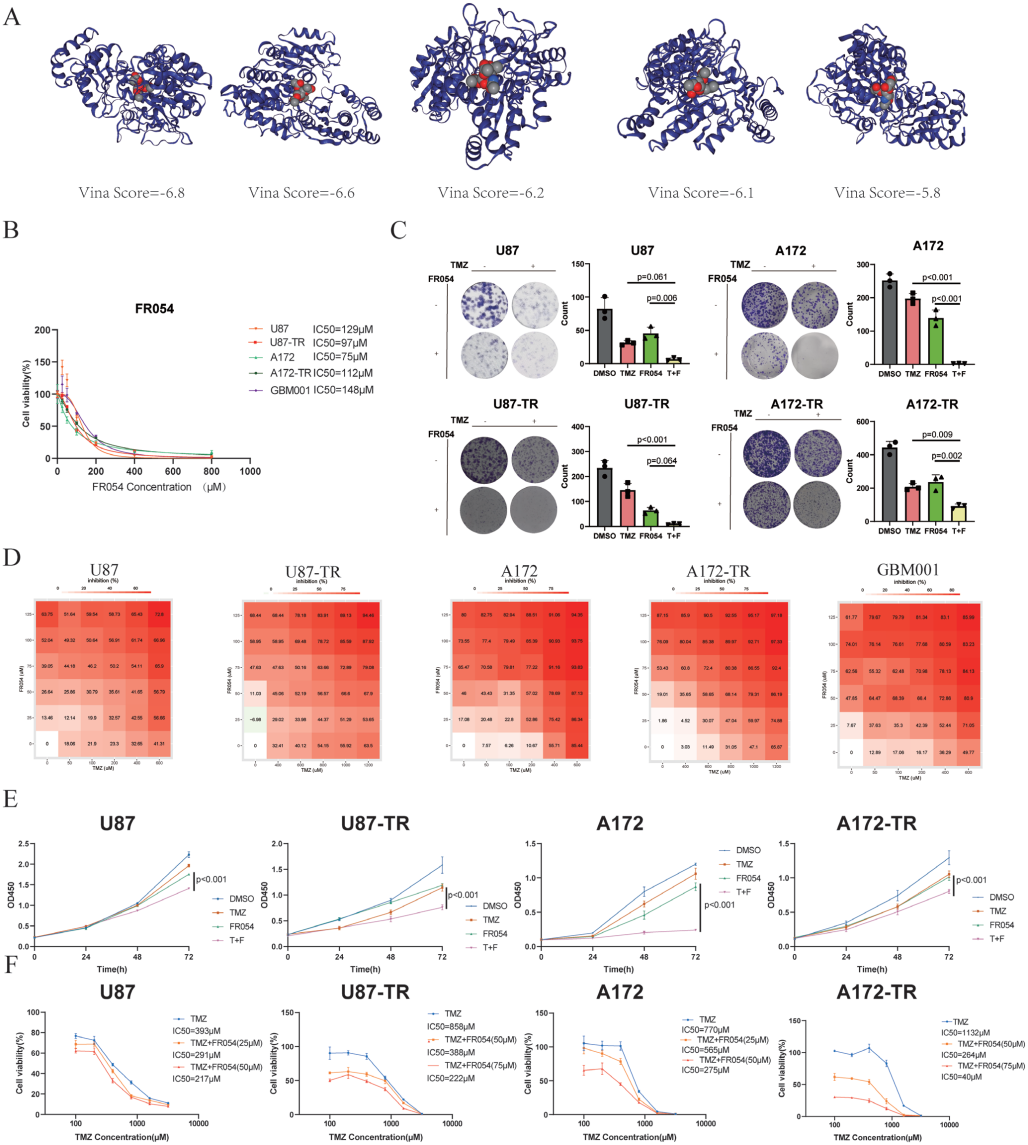
Combination therapy of TMZ and HBP Pathway Inhibitor FR054 displayed a significant synergistic effect on GBM cells. (A) Molecular docking of FR054 with PGM3 protein. Generated using CB‐Dock2, this model visualizes the potential binding sites of FR054 (spherical structures) to the PGM3 protein (in blue), providing insights into their interaction at the molecular level. (B) CCK8 assay IC50 curves for U87, U87‐TR, A172, A172‐TR, and GBM001 cells following 72‐h treatment with FR054. (C) Colony formation assay of U87, U87‐TR, A172, and A172‐TR cells after 14‐day drug treatment. Left: Representative colony images. Right: Quantitative analysis of colony numbers, normalized to untreated controls. (D) Heatmap of Bliss synergy indices for U87, U87‐TR, A172, and A172‐TR cells treated with varying concentrations of TMZ and FR054 for 72 h, calculated using SynergyFinder. Higher synergy scores indicate stronger synergistic effects. (E) OD450 values for U87, U87‐TR, A172, and A172‐TR cells treated with TMZ, FR054, or their combination for 72 h. Bliss index‐optimized concentrations were used to evaluate cell viability. Statistical comparisons were performed using Student's t‐test at 72 h. (F) IC50 curves of U87, U87‐TR, A172, and A172‐TR cells treated with FR054 in combination with TMZ at varying concentrations, assessed by CCK8 assay. These curves demonstrate the dose‐dependent reduction in TMZ IC50 values upon FR054 co‐treatment.

Molecular docking simulations using CB‐Dock2 identified five potential binding sites of FR054 with PGM3, all exhibiting Vina Scores below −5, indicating strong binding affinity (Figure 3A; Table S1). Furthermore, we evaluated the anti‐cancer and TMZ re‐sensitizing activity of FR054 in GBM cell lines, including TMZ‐resistant variants (U87, U87‐TR, A172, A172‐TR, and patient‐derived GBM001). CCK8 assays revealed differential sensitivity across the cell lines, with IC50 values of FR054 ranging from 75 μM (A172) to 148 μM (GBM001) after 72 h of treatment (Figure 3B). Colony formation assays demonstrated that combination therapy of TMZ and FR054 significantly enhanced growth inhibition compared to single‐agent treatments (Figure 3C).

The synergistic effects of FR054 and TMZ were assessed using the Bliss independence model via SynergyFinder. We selected the lowest concentrations of each drug under conditions where the Bliss synergy index exceeded 10 for further experiments. Significant synergy scores were observed at specific concentration combinations: U87 (Bliss synergy score = 10.77, 200 μM TMZ + 25 μM FR054), U87‐TR (Bliss synergy score = 14.28, 400 μM TMZ + 50 μM FR054), A172 (Bliss synergy score = 26.93, 200 μM TMZ + 25 μM FR054), A172‐TR (Bliss synergy score = 28.72, 800 μM TMZ + 50 μM FR054) and GBM001 (Bliss synergy score = 28.91, 200 μM TMZ + 25 μM FR054) (Figure 3D).

Time‐dependent growth inhibition was evaluated by measuring OD450 values at 0, 24, 48, and 72 h post‐treatment. Both FR054 alone and in combination with TMZ induced significant time‐dependent growth inhibition, with maximal effects observed at 72 h (Figure 3E; Figure S2A). Dose‐dependent effects were confirmed by analyzing TMZ IC50 shifts in the presence of FR054. In U87 cells, the TMZ IC50 decreased from 393 to 217 μM with increasing FR054 concentrations (25–50 μM). Similarly, in A172‐TR cells, the TMZ IC50 dropped from 1132 to 40 μM with FR054 (50–75 μM). These findings demonstrate the potent synergistic and dose‐dependent effects of FR054 in combination with TMZ (Figure 3F; Figure S2B). Collectively, these results highlight the therapeutic potential of FR054 as a novel adjunctive therapy for GBM, including TMZ‐resistant variants.

### 3.4 Combination Therapy of TMZ and FR054 Exhibited a Significant Inhibitory Effect on the Progression of GBM In Vivo and Organoid Models

**FIGURE 4:**
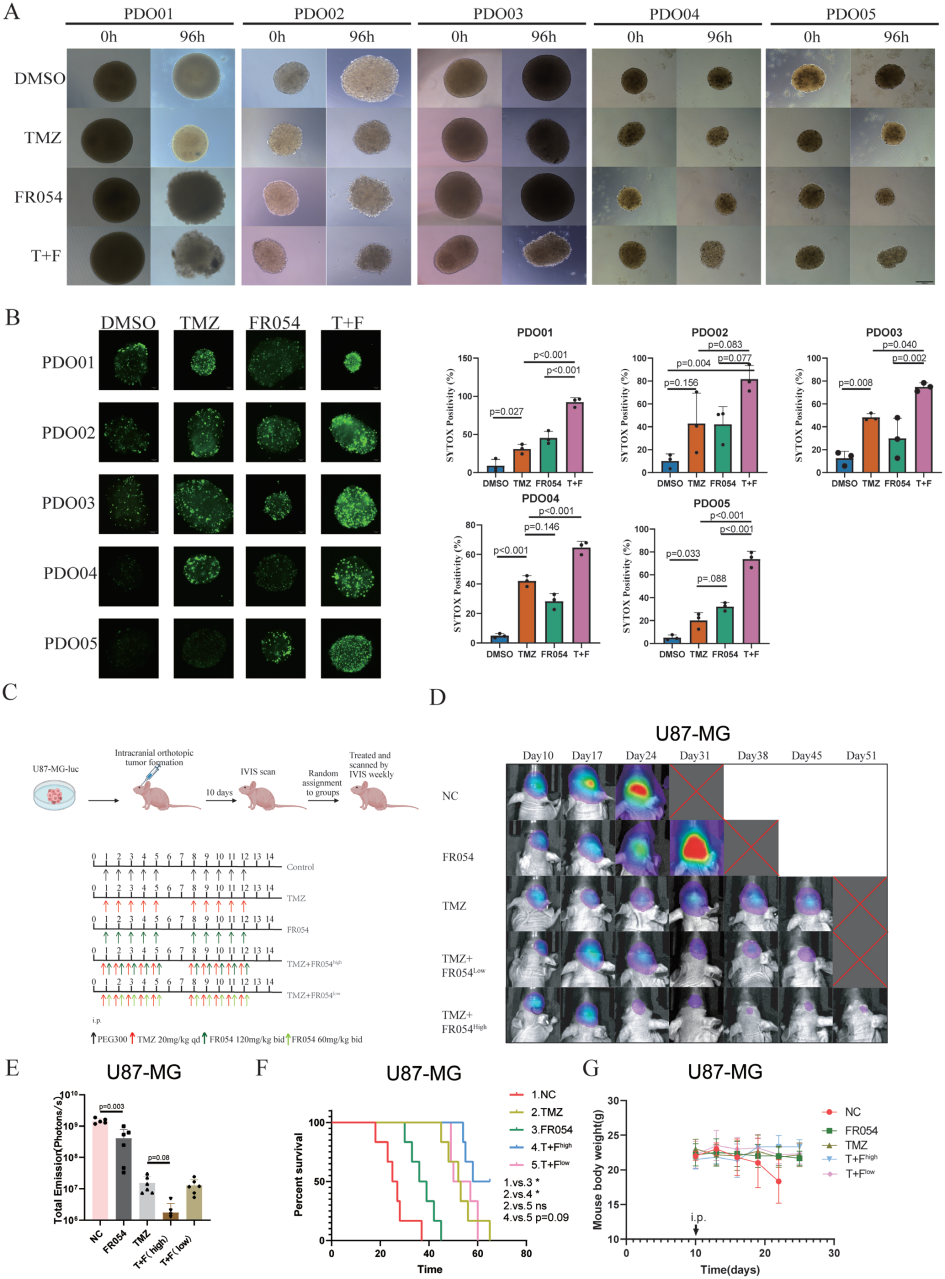
Combination therapy of ABX and FR054 exhibited a significant inhibitory effect on the progression of GBM in vivo and organoid models. (A) Morphological changes in organoids after 96‐h treatment with TMZ (1000 μM) and FR054 (50 μM), alone or in combination, observed under 100X magnification. (B) Fluorescence microscopy images of SYTOX Green‐stained organoids for detecting dead cells, along with quantification of SYTOX‐positive cells to assess cell death. Bar chart showing mean ± SD. Data were tested for normality using the Shapiro–Wilk test before performing one‐way ANOVA to compare means across groups (ns: *p* > 0.05). (C) Schematic representation of the tumor formation and treatment protocol in nude mice, randomly divided into five groups (*n* = 6 each): Control, TMZ monotherapy (20 mg/kg qd), FR054 monotherapy (120 mg/kg bid), low‐dose combination therapy (TMZ 20 mg/kg qd + FR054 60 mg/kg bid), and high‐dose combination therapy (TMZ 20 mg/kg qd + FR054 120 mg/kg bid). (D) In vivo bioluminescence imaging of tumor growth every 7 days post‐inoculation in each group (*n* = 6). (E) Bioluminescence imaging and quantification of fluorescence intensity after 14 days of treatment. (F) Kaplan–Meier survival curves for nude mice in each treatment group. (G) Body weight changes monitored every 3 days during treatment.

To further validate the synergistic therapeutic effects of FR054 and TMZ, we utilized patient‐derived organoid models treated with TMZ (1000 μM) and FR054 (50 μM) for 96 h. Optical microscopy observations (Figure 4A) revealed significant morphological changes in organoids treated with combination therapy compared to monotherapy groups and the DMSO control, indicating a stronger inhibitory or destructive effect by the combined treatment. For quantification of cell death, SYTOX Green staining was performed after 96 h of drug exposure and observed under fluorescence microscopy (Figure 4B). The results showed a marked increase in SYTOX Green‐positive cells in the combination group compared to monotherapy groups, confirming the enhanced cytotoxic effect of FR054 and TMZ in the organoid model. We summarized the clinical and molecular characteristics of five patients, including age, gender, molecular profiles, and diagnosis (Table S2).

To investigate these synergistic antitumor effects in vivo, an orthotopic GBM model was established by injecting Fluc‐expressing U87 cells into the brains of nude mice. Bioluminescence imaging on Day 10 confirmed tumor establishment, and mice were randomized into five treatment groups: negative control (NC), TMZ monotherapy (20 mg/kg, qd), high‐dose FR054 monotherapy (FR054^high^, 120 mg/kg, bid), combination therapy with TMZ and high‐dose FR054 (TMZ + FR054^high^), and low‐dose FR054 (TMZ + FR054^low^, 60 mg/kg, bid). Treatments were administered via intraperitoneal injection using a solvent mixture (50% PEG300 and 50% saline) (Figure 4C). Bioluminescence imaging every 7 days monitored tumor progression (Figure 4D), with fluorescence intensity quantified for analysis (Figure 4E). Both FR054 and TMZ monotherapies significantly reduced tumor fluorescence intensity compared to NC (*p* < 0.001). Notably, the TMZ + FR054^high^ group showed a marginally significant reduction in tumor fluorescence intensity compared to TMZ alone (*p* = 0.08). In contrast, no significant difference was observed between the TMZ + FR054^low^ and TMZ monotherapy groups.

Survival analysis (Figure 4F) mirrored the trends seen in fluorescence intensity. While both FR054 and TMZ monotherapies effectively suppressed tumor growth, the TMZ + FR054^high^ combination demonstrated a more pronounced therapeutic benefit. However, the TMZ + FR054^low^ combination did not enhance efficacy over TMZ alone. Body weight monitoring every 3 days indicated no significant differences among groups (Figure 4G), suggesting minimal adverse effects from the treatment regimens on overall mouse health.

### 3.5 Synergistic Anti‐Tumor Effects of FR054 and TMZ on GBM Cells Through Downregulating Protein O‐GlcNAcylation and Activating Ferroptosis

**FIGURE 5:**
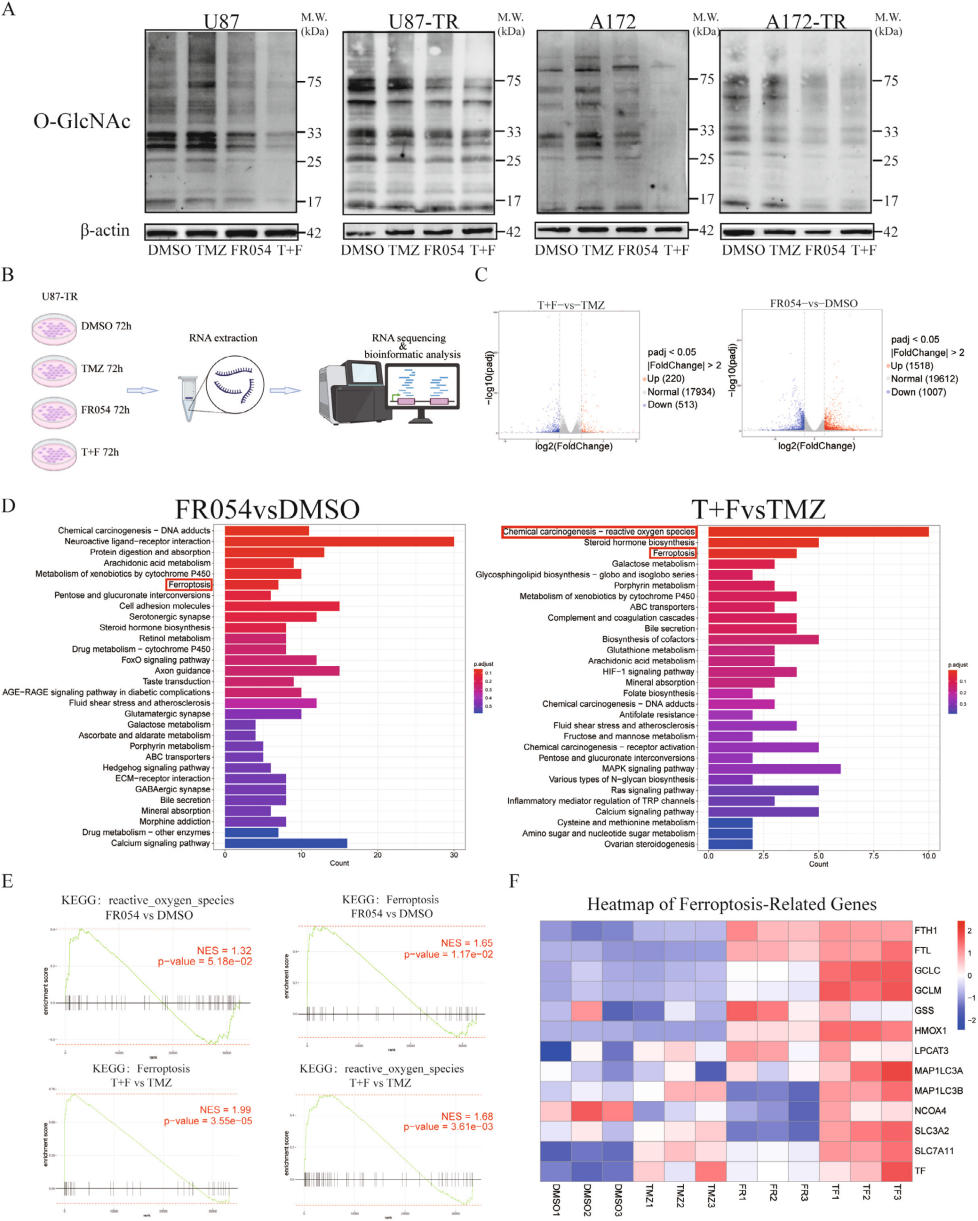
Synergistic anti‐tumor effects of FR054 and TMZ on glioblastoma cells through downregulating protein O‐GlcNAcylation and activating ferroptosis. (A) Western blot analysis of O‐GlcNAc levels in U87, U87‐TR, A172, and A172‐TR cells after 72‐h treatments with TMZ, FR054, or their combination. (B) Transcriptional profiling of U87‐TR cells treated for 72 h with DMSO, TMZ, FR054, or TMZ + FR054; RNA sequencing was performed to identify differentially expressed genes (DEGs). (C) Volcano plots illustrating differentially expressed genes (DEGs) between the TMZ + FR054 vs. TMZ and FR054 vs. DMSO groups (|log2FC| > 1, *P*adj < 0.05). (D) Bar graph depicting KEGG pathway enrichment analysis for the FR054 vs. DMSO comparison, ordered by statistical significance. (E) Gene Set Enrichment Analysis (GSEA) focusing on ferroptosis‐ and ROS‐related pathways for upregulated genes in the TMZ + FR054 vs. TMZ and FR054 vs. DMSO groups. (F) Heatmap displaying the expression levels of ferroptosis‐promoting genes across four experimental groups.

Due to FR054 being an HBP pathway inhibitor targeting PGM3, the HBP pathway plays a critical role in protein glycosylation, including O‐GlcNAcylation. Therefore, we evaluated the impact of TMZ and FR054, either alone or in combination for 72 h, on protein O‐GlcNAcylation in each group using Western blot analysis (Figure 5A). The results revealed a significant decrease in protein O‐GlcNAcylation in GBM cells treated with FR054. Moreover, the combined treatment group showed an even more pronounced reduction in protein O‐GlcNAcylation, suggesting that FR054 may exert its anti‐tumor effects through downregulating protein O‐GlcNAcylation.

To further investigate other underlying mechanisms of the synergistic effects of FR054 and TMZ on GBM cells apart from protein O‐GlcNAcylation, we performed transcriptome sequencing analysis in four treatment groups: DMSO control, 600 μM TMZ alone, 50 μM FR054 alone, and a combination of FR054 and TMZ (T + F group) (Figure 5B). Differential gene expression analysis identified a total of 220 upregulated and 513 downregulated genes in the T + F group compared to the TMZ group, while 1518 upregulated and 1007 downregulated genes were detected in the FR054 vs. DMSO group (Figure 5C). KEGG pathway enrichment analysis indicated that differential genes in the T + F versus TMZ group were also enriched in chemical carcinogenesis–ROS pathways and ferroptosis pathways (Figure 5D), suggesting these pathways might play a pivotal role in enhancing temozolomide sensitivity by FR054. Gene Set Enrichment Analysis (GSEA) was performed to further explore the molecular mechanisms underlying this synergy. Specifically, GSEA analysis between the dual‐drug group and TMZ alone, as well as between FR054 alone and DMSO, revealed significant enrichment scores (NES) and *p*‐values for ferroptosis and ROS pathways (Figure 5E). The correlation between FR054's effect and ferroptosis pathways was further investigated using a heatmap based on iron death‐related genes from the FerrDb database (Figure 5F). It was observed that iron death‐related genes were significantly upregulated in the TMZ and FR054 combination therapy group compared to the control groups, indicating that the combination therapy may enhance tumor cell killing by activating ferroptosis pathways.

**FIGURE 6:**
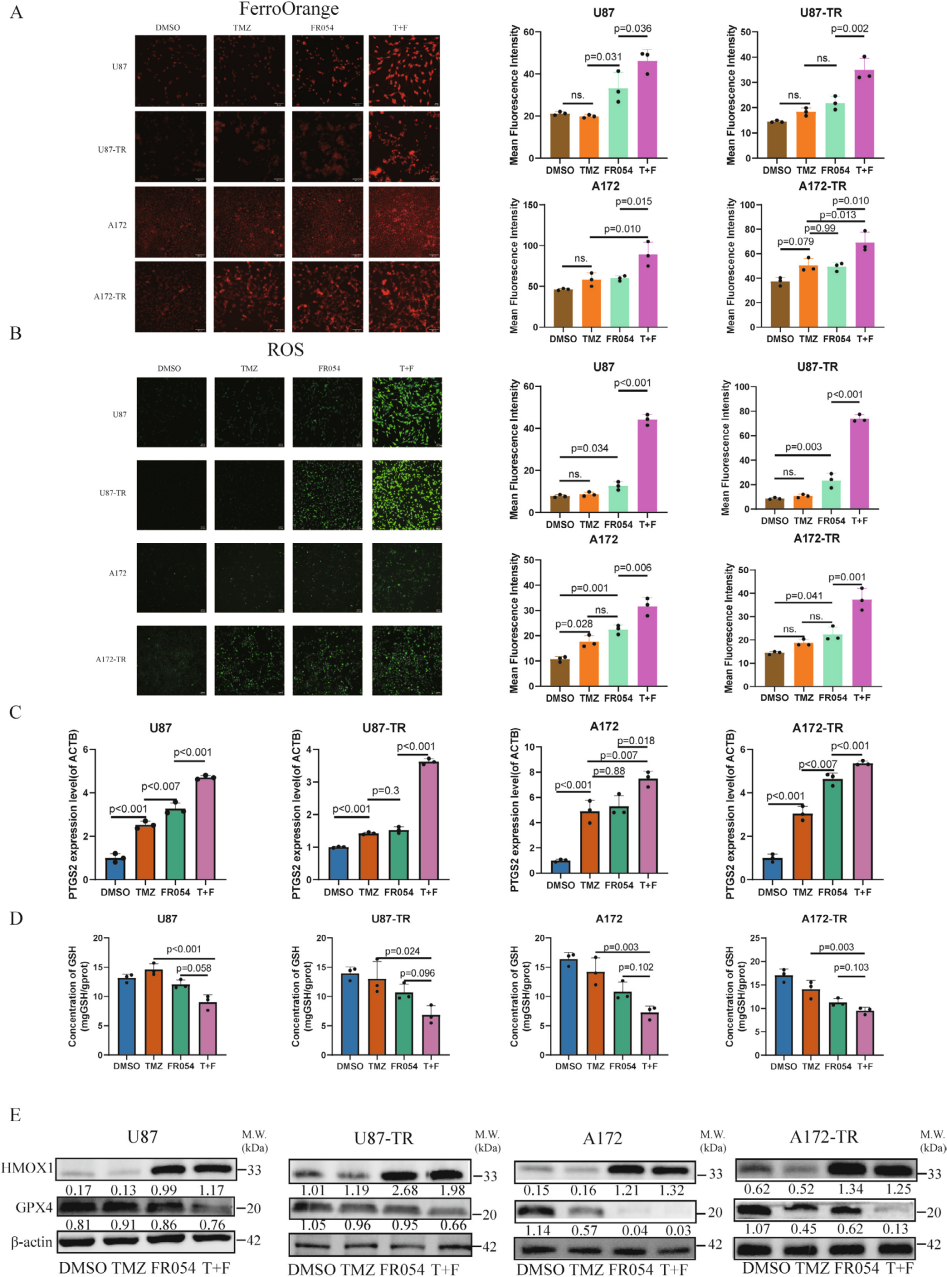
Synergistic anti‐tumor effects of FR054 and TMZ on glioblastoma cells through ferroptosis activation by regulating HMOX1 and GPX4 expression levels. (A) Fluorescence microscopy images and quantification of FerroOrange‐stained GBM cells after 72‐h treatments, used to evaluate intracellular ferrous iron levels (*n* = 3). ns: *p* > 0.05. (B) FITC fluorescence microscopy images and quantification of DCFH‐DA‐stained GBM cells following treatments, measuring average fluorescence intensity as an indicator of reactive oxygen species (ROS) levels (*n* = 3). ns: *P* > 0.05. (C) qPCR analysis of PTGS2 mRNA expression in GBM cells post‐treatment. (D) Relative glutathione (GSH) levels in GBM cells after 72‐h treatments, normalized to untreated controls. Data presented as mean ± SD. (E) Western blot analysis of ferroptosis‐related proteins HMOX1 and GPX4 in U87 and U87‐TR cells following 72‐h treatments. Bar chart showing mean ± SD. Data were tested for normality using the Shapiro–Wilk test prior to one‐way ANOVA for comparing means across groups (ns: *p* > 0.05).

Further, we performed validation experiments to confirm the underlying mechanism. FerroOrange and DCFH‐DA assays revealed significantly increased intracellular iron ion levels and ROS levels in the FR054 and TMZ combination treatment group compared with other groups (Figure 6A,B). Moreover, qRT‐PCR confirmed a substantial upregulation of PTGS2 mRNA—a recognized marker of ferroptosis—in the combination treatment group compared to other groups (Figure 6C). Additionally, GSH levels were quantified using a GSH assay kit, which indicated a notable reduction in GSH content in GBM cells receiving the combined therapy of TMZ and FR054 compared to other groups (Figure 6D).

Additionally, due to increasing evidence confirming the significant roles of GPX4 and HMOX1 proteins in inducing ferroptosis, western blotting was performed to examine the expression levels of GPX4 and HMOX1 proteins. The results revealed a significant downregulation of GPX4 expression and a significant upregulation of HMOX1 expression in GBM cells treated with combination therapy compared to other groups (Figure 6E).

## 4 Discussion

In the present study, we investigated novel therapeutic strategies to overcome TMZ resistance in GBM, a major obstacle in the clinical management of this aggressive malignancy. Although the combination of TMZ with radiotherapy has demonstrated significant survival benefits for GBM patients, the emergence of TMZ resistance remains a critical challenge, often leading to tumor recurrence and treatment failure. Therefore, identifying small‐molecule compounds capable of crossing the blood–brain barrier and synergizing with TMZ has become a focal point in GBM research.

To mimic the clinical scenario of TMZ resistance, we established TMZ‐resistant GBM cell lines and performed proteomic and bioinformatic analyses. These analyses revealed an upregulation of the HBP in TMZ‐resistant cells. Western blot experiments further confirmed increased O‐GlcNAcylation levels in GBM cells after 72 h of TMZ treatment, with even higher levels observed in TMZ‐resistant cells compared to their parental counterparts. This finding suggests that chronic TMZ exposure may induce the upregulation of HBP‐related genes, thereby increasing UDP‐GlcNAc production, which serves as the substrate for O‐GlcNAcylation. Elevated O‐GlcNAcylation levels may subsequently promote TMZ resistance in GBM cells. Consequently, targeting the HBP pathway to reduce cellular UDP‐GlcNAc levels could represent a promising strategy to mitigate TMZ resistance.

Our bioinformatic analysis identified PGM3, a crucial enzyme in HBP, as being associated with poor prognosis in GBM patients. Building on previous reports demonstrating the antitumor efficacy of FR054—a competitive inhibitor of PGM3—in breast cancer, pancreatic cancer, and pancreatic ductal adenocarcinoma—we extended these findings to GBM [11, 12, 13]. Our study demonstrates the synergistic effects of FR054 and TMZ across various experimental models, including GBM cell lines, organoids, and animal models, without observable toxic side effects. Mechanistically, FR054 enhances GBM sensitivity to TMZ by reducing O‐GlcNAcylation levels and promoting ferroptosis. While direct validation of FR054's impact on UDP‐GlcNAc levels was not performed in our research, this relationship has been reported in prior studies [11]. Additionally, we observed the upregulation of ferroptosis‐related genes, such as NQO1 and HMOX1, in transcriptomic sequencing data following FR054 treatment. These findings align with previous reports by Chen et al. [37], who demonstrated that inhibition of OGT using 5SGlcNAc reduced KEAP1 O‐GlcNAcylation at serine 104, leading to decreased NRF2 ubiquitination, enhanced NRF2 nuclear translocation, and subsequent upregulation of downstream genes like NQO1 and HMOX1, which are now recognized as ferroptosis‐promoting genes. Based on these observations, we propose that FR054 may induce ferroptosis in GBM cells through the KEAP1‐NRF2‐HMOX1 axis. However, additional experimental validation is required to confirm this hypothesis. In the realm of TMZ resistance in GBM, recent studies have begun to uncover a correlation between ferroptosis and TMZ resistance [14]. Ferroptosis, an iron‐dependent form of programmed cell death characterized by lipid peroxidation, has been implicated in the response of cancer cells to various therapies [38], including TMZ. However, while these associations are emerging, the precise mechanisms underlying how ferroptosis contributes to TMZ resistance remain poorly understood.

The role of O‐GlcNAcylation in cancer biology has been extensively studied in recent years [8]. Most studies have focused on modulating O‐GlcNAcylation by targeting OGT or OGA using inhibitors such as OSMI‐1 [39], 5SGlcNAc [40], or Thiamet‐G [41]. In the context of GBM, Lorela Ciraku et al. [42] demonstrated the association between O‐GlcNAcylation and GBM cell proliferation through OGT knockdown and 5SGlcNAc treatment. However, the relationship between the HBP pathway, its end product UDP‐GlcNAc, and GBM remains underexplored. While studies in other cancers have primarily focused on GFPT1 [43], the rate‐limiting enzyme of the HBP, the role of PGM3 has been largely overlooked. Notably, although PGM3 catalyzes a reversible reaction within the HBP, recent evidence suggests that PGM3 knockdown [10] or inhibition significantly [11] reduces tumor cell proliferation and decreases O‐GlcNAcylation levels. Our findings with FR054 corroborate these observations, highlighting the critical role of PGM3 in the HBP and its potential as a therapeutic target.

Despite the promising results presented in this study, several limitations should be acknowledged. Firstly, although we observed decreased protein O‐GlcNAcylation levels and induction of ferroptosis following FR054 treatment, the precise molecular mechanisms linking FR054‐mediated changes in O‐GlcNAcylation to ferroptosis remain to be fully elucidated. Further mechanistic studies focusing on key regulatory pathways such as Nrf2/HMOX1 and GPX4 signaling are warranted to better understand this interaction. Secondly, while patient‐derived organoid models were employed to recapitulate human GBM biology, the sample size was relatively small. Expanding the number of organoids derived from genetically heterogeneous backgrounds will enhance the robustness and generalizability of our findings. Moreover, more comprehensive in vivo studies using orthotopic xenograft models are necessary to evaluate the safety profile and therapeutic efficacy of FR054 in a physiologically relevant context. Finally, our findings suggest that combining FR054 with TMZ may represent a novel and effective strategy for overcoming TMZ resistance in glioblastoma. However, additional preclinical investigations—including detailed pharmacokinetic and toxicity assessments—are essential before advancing this combination therapy into clinical trials. Of note, FR054 administered via systemic routes may exhibit limited ability to cross the blood–brain barrier (BBB), potentially restricting its therapeutic efficacy in brain tumors. Therefore, future research should focus on developing nanomaterial‐based drug delivery systems to improve the targeted delivery and brain penetration of FR054. Such strategies could significantly enhance its bioavailability and therapeutic potential in the treatment of glioblastoma [44, 45, 46].

Our study offers novel insights into the role of the HBP pathway in TMZ resistance and emphasizes the therapeutic potential of targeting PGM3 with FR054 to enhance TMZ efficacy in GBM. These findings highlight the significance of investigating metabolic pathways as a strategy to overcome drug resistance and improve clinical outcomes for GBM patients. Future studies should aim to elucidate the molecular mechanisms underlying FR054‐induced ferroptosis and validate its efficacy across broader preclinical models and clinical trials.

## 5 Conclusion

Our study demonstrated that TMZ‐resistant GBM cells exhibit upregulated hexosamine biosynthesis and O‐GlcNAcylation, with high PGM3 expression correlating with poor prognosis. The combination of FR054 and TMZ treatment exhibited potent synergistic effects in multiple models, including patient‐derived organoids and orthotopic GBM mouse models. Targeting the HBP pathway with FR054 can overcome TMZ resistance in GBM by reducing O‐GlcNAc modification levels and inducing ferroptosis. This novel therapeutic approach enhances the efficacy of TMZ, providing a promising strategy for GBM patients with limited treatment options.

## Author Contributions

Conceptualization: Rongxu Ye and Guo‐zhong Yi; methodology: Rongxu Ye and Guo‐zhong Yi; software: Rongxu Ye, Wanghao Zhang, and Junyi Xu; validation: Rongxu Ye, Wanghao Zhang, and Huayang Zhang; formal analysis: Rongxu Ye; investigation: Rongxu Ye, Wanghao Zhang, and Huayang Zhang; resources: Rongyang Xu and Ye Zhu; data curation: Rongxu Ye; writing – original draft preparation: Rongxu Ye; writing – review and editing: Guo‐zhong Yi; visualization: Rongxu Ye and Wanghao Zhang; supervision: Xi‐an Zhang and Guo‐zhong Yi; project administration: Xi‐an Zhang, Guanglong Huang, and Guo‐zhong Yi; funding acquisition: Xi‐an Zhang, Guanglong Huang, and Guo‐zhong Yi. All authors have read and agreed to the published version of the manuscript.

## Conflicts of Interest

The authors declare no conflicts of interest.

## Supporting information


**Figure S1:** cns70546‐sup‐0001‐Figures.docx.

**Table S1:** cns70546‐sup‐0002‐TableS1.xlsx.

**Table S2:** cns70546‐sup‐0003‐TableS2.docx.

**Data S1:** cns70546‐sup‐0004‐DataS1.docx.

## Data Availability

The data that support the findings of this study are available from the corresponding authors upon reasonable request.
