# Supplementary material for: The HBP Pathway Inhibitor FR054 Enhances Temozolomide Sensitivity in Glioblastoma Cells by Promoting Ferroptosis and Inhibiting O‐GlcNAcylation
Source: CNS Neurosci Ther. 2025 Aug 7;31(8):e70546. doi: 10.1111/cns.70546 (PMC12329428; doi:10.1111/cns.70546)
Supplement: Supplementary file 1 — Figure S1: cns70546‐sup‐0001‐Figures.docx. [file CNS-31-e70546-s002.docx]

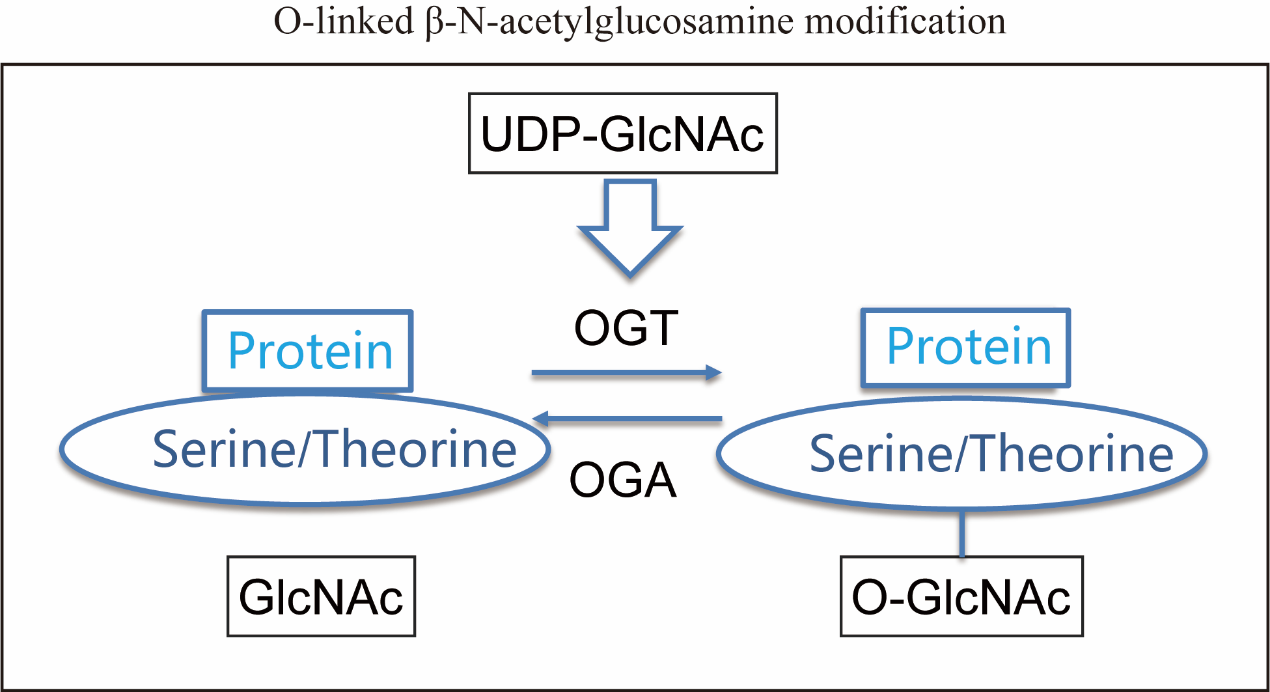


**Figure S1. Proteomics analysis reveals enrichment of the hexosamine biosynthesis pathway and upregulation of O-GlcNAcylation in temozolomide-resistant cells.**

Schematic Representation of Protein O-GlcNAcylation and De-O-GlcNAcylation.


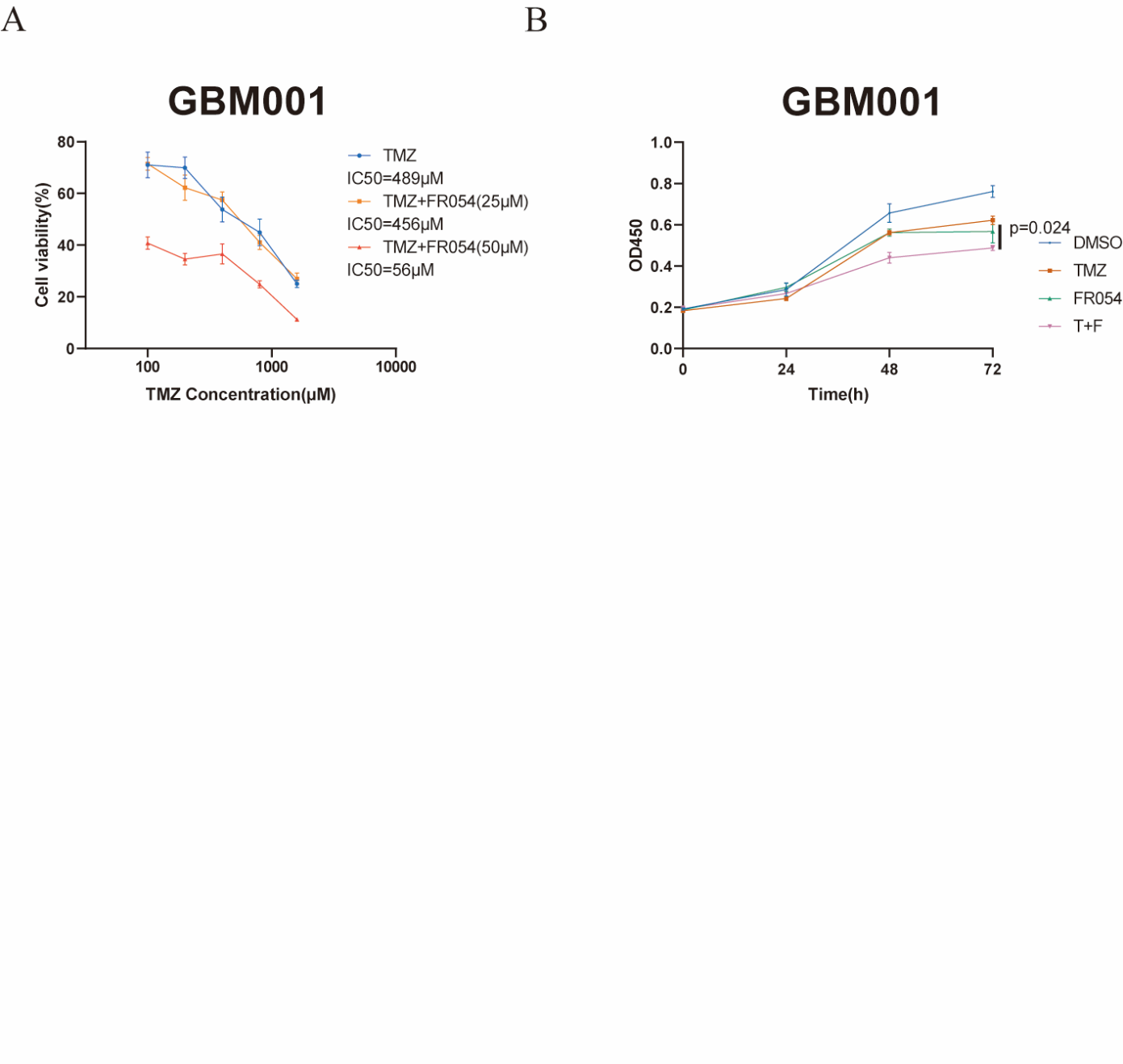


**Figure S2. Synergistic Inhibition of Tumor Growth by HBP Pathway Inhibitor FR054 in Combination with Temozolomide In Vitro.** (A) IC50 curves of GBM001 cells treated with FR054 in combination with TMZ at varying concentrations, assessed by CCK8 assay. (B) OD values at 450 nm for GBM001 cells treated with TMZ, FR054, or their combination for 72 hours. The Bliss index-optimized concentrations were used to evaluate cell viability.
