# Supplementary material for: The HBP Pathway Inhibitor FR054 Enhances Temozolomide Sensitivity in Glioblastoma Cells by Promoting Ferroptosis and Inhibiting O‐GlcNAcylation
Source: CNS Neurosci Ther. 2025 Aug 7;31(8):e70546. doi: 10.1111/cns.70546 (PMC12329428; doi:10.1111/cns.70546)
Supplement: Supplementary file 3 — Table S2: cns70546‐sup‐0003‐TableS2.docx. [file CNS-31-e70546-s004.docx]

**Table S2. Clinical and Molecular Characteristics of Five GBM Patients Included in the PDO Construction**

| **No.** | **Age** | **Gender** | **IHC Information** | **Diagnosis** |
| --- | --- | --- | --- | --- |
| PDO01 | 57 | Female | GFAP(+)、Oligo-2(+)、IDH1(-)、ATRX(+)、P53（Strongly positive+，90%，mutant pattern）、BRAF-V600E(一)、CD34(-)、C-met (0)、MGMT (+，45%)、Ki-67(+，90%) | **Glioblastoma, IDH1 wild-type** |
| PDO02 | 54 | Female | GFAP(+)、oligo-2(+)、P53（**Variable Intensity**+，about 50%)、IDH1(-)、ATRX(+)、BRAF-V600E （-)、CD34（vascular+)、C-met (-)、EMA(+)、Neu-N（+)、S-100(+)、H3K27M（-)、H3K27Me3(+)、Ki-67(+，about 70%) | **Glioblastoma, IDH1 wild-type** |
| PDO03 | 59 | Male | GFAP(+)、oligo-2(+)、P53（**Variable Intensity**+，**wild-type**)、IDH1(-)、ATRX(+)、BRAF-V600E（-）、C-met(-)、CD34（vascular+)、Ki-67(+，about 60%) | **Glioblastoma, IDH1 wild-type** |
| PDO04 | 34 | Female | GFAP(+)、oligo-2(+)、P53(**Variable Intensity**+，about60%)、IDH1(+)、ATRX(+)、BRAF-V600E(-)、CD34 (vascular+)、C-met(-)、H3K27M(-)、H3K27Me3(+)、Ki-67(+，about5%): | **Adult-type diffuse astrocytoma**  **IDH-mutant type** |
| PDO05 | 50 | Female | GFAP(+)、o1igo-2(+)、Neu-N(-)、IDH1(-)、ATRX(+)、P53(-)、BRAF-V600E(-)、C-met(0)、CD34(vascular+)、H3K27M(-)、H3K27Me3(+)、Ki-67(+,about35%) | **Glioblastoma, IDH1 wild-type** |
