# Supplementary material for: The HBP Pathway Inhibitor FR054 Enhances Temozolomide Sensitivity in Glioblastoma Cells by Promoting Ferroptosis and Inhibiting O‐GlcNAcylation
Source: CNS Neurosci Ther. 2025 Aug 7;31(8):e70546. doi: 10.1111/cns.70546 (PMC12329428; doi:10.1111/cns.70546)
Supplement: Supplementary file 4 — Data S1: cns70546‐sup‐0004‐DataS1.docx. [file CNS-31-e70546-s001.docx]

**Cell Viability Assay**

Cell viability was assessed using the Cell Counting Kit-8 (CCK-8) assay. Cells were seeded in 96-well plates at a density of 1 × 10³ cells per well, with six replicate wells for each group. After culturing for 24 hours to allow cell attachment, the cells were treated with the specified drugs for 72 hours. Following treatment, cell viability was evaluated using the CCK-8 kit (New Cell & Molecular Biotech, C6005) according to the manufacturer’s instructions. Briefly, 10 µL of CCK-8 solution was added to each well, and the plates were incubated at 37°C for an additional 2 hours. Absorbance was measured at 450 nm using a microplate reader to determine cell viability.

**Colony Formation Assay**

The colony formation assay was performed to evaluate the long-term proliferative potential of GBM cells. Cells were seeded in six-well plates at a density of 500 cells per well and treated with the specified drugs for 24 hours. After the treatment period, the culture medium was replaced with fresh growth medium. The cells were then incubated for an additional two weeks to allow colony formation. At the end of the incubation period, visible colonies were fixed with 100% methanoland subsequently stained with 0.1% crystal violet dissolved in 20% methanol for 15 minutes. The number of colonies was quantified using ImageJ software (version 1.48, National Institutes of Health, USA). This assay provides a reliable measure of clonogenic survival under different experimental conditions.

**LC-MS/MS analysis**

For sample lysis and protein extraction, SDT buffer (4% SDS, 100 mM Tris-HCl, 1 mM DTT, pH 7.6) was utilized, followed by quantification of the extracted proteins using the BCA Protein Assay Kit (Bio-Rad, USA). Proteins were enzymatically digested following the filter-aided sample preparation (FASP) procedure described by Matthias Mann. LC-MS/MS analysis was performed on a Q Exactive mass spectrometer (Thermo Scientific) coupled with an Easy nLC system (Proxeon Biosystems, now Thermo Fisher Scientific) over gradient durations of 60, 120, or 240 minutes. Raw MS data from each sample were combined and analyzed for peptide identification and quantification using MaxQuant software version 1.5.3.17, enabling comprehensive qualitative and quantitative proteomic profiling.

**RNA-Seq**

Total RNA was extracted from tissue samples, quantified using Nanodrop 2000, and its integrity assessed by agarose gel electrophoresis and Agilent 2100 Bioanalyzer (RIN ≥ 7). mRNA was enriched using Oligo(dT) magnetic beads, fragmented (~300 bp) with fragmentation buffer, and purified using magnetic beads. First-strand cDNA was synthesized using random hexamer primers and reverse transcriptase, followed by second-strand synthesis. Double-stranded cDNA underwent end-repair, A-tailing, and adaptor ligation. Libraries were PCR-amplified for 15 cycles, size-selected on a 2% agarose gel, and quantified using TBS-380 (Picogreen). Finally, libraries were pooled and sequenced on the Illumina NovaSeq 6000 platform (PE 2 × 150 bp) after cluster generation on a cBot system.

**qRT-PCR**

Total RNA was extracted using the RNeasy Kit (Foregene) and reverse-transcribed with the SuperScript II kit (Takara) using random primers. Real-time PCR was performed using SYBR Green Master Mix (Takara) on the QuantStudio 6 Flex system. The ΔΔCT method was applied for relative quantification, with reactions run in triplicate. Melting curve analysis confirmed amplification specificity. Primers used were: PTGS2 (F:5’-CGGTGAAACTCTGGCTAGACAG-3’ ,R:5’-GCAAACCGTAGATGCTCAGGGA-3’) and ACTB (F: 5’-CCTGGCACCCAGCACAAT-3’, R: 5’-GGGCCGGACTCGTCATAC-3’), with BACT serving as the internal control.

**Western Blot analysis**

For Western blot analysis, cells were first lysed using a lysis buffer (containing protease and phosphatase inhibitors) to extract total protein. Protein concentrations were determined using the Bicinchoninic Acid (BCA) Assay Kit according to the manufacturer's instructions. Equal amounts of protein (typically 20-30 μg per lane) were separated by Sodium Dodecyl Sulfate-Polyacrylamide Gel Electrophoresis (SDS-PAGE) and then electrotransferred onto Polyvinylidene Fluoride (PVDF) membranes. After blocking with 5% non-fat milk in Tris-buffered saline containing 0.1% Tween-20 (TBST) for 1 hour at room temperature, the membranes were incubated overnight at 4°C with primary antibodies specific to the proteins of interest. Following extensive washing with TBST, the membranes were incubated with horseradish peroxidase-conjugated secondary antibodies for 1 hour at room temperature. Protein bands were visualized using enhanced chemiluminescence (ECL) detection reagents and exposed to X-ray film or captured using a ChemiDoc imaging system. β-actin was used as an internal control to normalize protein loading across different lanes. Densitometric analysis was performed using ImageJ software (version 1.48, National Institutes of Health, USA) to quantify the relative expression levels of target proteins.

The primary antibodies were anti-GPX4 (Cat.no. 52455T; Cell Signaling), anti-HMOX1 (Cat.no. T55113F; abmart), anti-O-GlcNAc (Cat.no. ma1-072; Thermofisher), anti-β-actin (Cat.no. AF5003, Beyotime).
